# The Predictive Validity of the Return-to-Work Self-Efficacy Scale for Return-to-Work Outcomes in Claimants with Musculoskeletal Disorders

**DOI:** 10.1007/s10926-015-9580-7

**Published:** 2015-05-20

**Authors:** Sandra Brouwer, Benjamin C. Amick, Hyunmi Lee, Renée-Louise Franche, Sheilah Hogg-Johnson

**Affiliations:** Department of Health Sciences, Community and Occupational Medicine, University Medical Center Groningen, University of Groningen, Antonius Deusinglaan 1, Building 3217, Room 620, 9713 AV Groningen, The Netherlands; Institute for Work & Health, Toronto, ON Canada; Robert Stempel College of Public Health and Social Work, Department of Health Policy and Management, Miami, FL USA; WorkSafe BC, Vancouver, BC Canada; Simon Fraser University, Vancouver, BC Canada; University of British Columbia, Vancouver, BC Canada; Dalla Lana School of Public Health, University of Toronto, Toronto, ON Canada

**Keywords:** Self-efficacy, Return-to-work, Predictive validity, Questionnaire, Improvement, Musculoskeletal, Pain, Disability, Psychiatric

## Abstract

*Purpose* To examine the predictive validity of the Return-to-Work Self-Efficacy (RTWSE) Scale in terms of the scale’s baseline absolute values and of changes in self-efficacy scores, with the outcome of return-to-work (RTW) status in a sample of injured workers with upper extremity and back musculoskeletal disorders. *Methods* RTWSE was measured with a 10-item scale assessing Overall RTWSE and three self-efficacy subdomains: (1) ability to cope with pain, (2) ability to obtain help from supervisor and (3) ability to obtain help from co-workers. Outcome measures included RTW status (yes/no) measured at 6- and 12-month follow-up. RTWSE improvement was defined as an increase in self-efficacy scores between baseline and 6-month follow-up time points. Logistic regression analyses were performed with RTW status as the dependent variable and adjusted for age, gender, educational level, personal income, pain site, pain severity, functional status, and depressive symptoms, and for baseline RTWSE scores in the improvement score analyses. *Results* A total of 632 claimants completed the baseline telephone interview 1 month post-injury; 446 subjects completed the 6-month interview (71 %) and 383 subjects completed the 12-month interview (61 %). The baseline Pain RTWSE scores were found to be useful to predict RTW status 6 months post-injury, with a trend for baseline Overall RTWSE. Improvements over time in Overall RTWSE and in Co-worker RTWSE were found to be useful to predict 12-month RTW status, with trends for improvements in Supervisor RTWSE and Pain RTWSE. *Conclusion* The study found evidence supporting the predictive validity of the RTWSE scale within 12 months after injury. The RTWSE scale may be a potentially valuable scale in research and in managing work disabled claimants with musculoskeletal disorders.

## Introduction

For the majority of workers with musculoskeletal (MSK) pain who are off work, return to work occurs within the first 3 months following the onset of a work disability episode [1, 2]. However, in some individuals, MSK pain progresses into chronic disability, even though no important physical changes may be detected [3]. Cognitions pertaining to perceived control play a major role in the adjustment of individuals with chronic pain [4–6]. One important control-related construct is self-efficacy; one’s belief that one can perform a specific behaviour successfully [7]. Self-efficacy has been found to be an important factor in pain control, adaptive psychological functioning, physical performance and disability management [4, 8–10]. Self-efficacy has proven useful in understanding and facilitating return-to-work (RTW) behaviour [11–16].

According to Bandura [7, 17], self-efficacy is highly predictive of the initiation and persistent execution of behaviour. Highly self-efficacious individuals set themselves more challenging goals, invest more to pursue these goals, persist longer and are better at dealing with setbacks than persons with lower self-efficacy. There is consensus in the field that context-specific measures of self-efficacy are more appropriate operationalization of the construct as described in social learning theory. Within the context of RTW, people with low self-efficacy for returning to work could be expected to postpone their return to work and to be less successful in their attempts to return to work than employees with higher levels of self-efficacy.

Self-efficacy has predictive validity for a range of work-related behaviours, such as RTW for employees with physical disabilities [18, 19] and work resumption of unemployed individuals with mental health problems [20, 21]. In a community-based cohort of workers undergoing carpal tunnel surgery, worsening self-efficacy between preoperative assessment and 2-month follow-up was associated with work absence at 6 months [14]. Improved self-efficacy predicted 6-month successful work role functioning [22] after adjusting for baseline self-efficacy scores. These findings show that evaluating both baseline and change scores might be of potential relevance in research in the RTW context. Improved knowledge about the predictive validity of self-efficacy within the context of RTW could assist clinicians and case managers. Baseline self-efficacy scores can be helpful in identifying early on in a claim those workers who are at high risk of prolonged work absence and who are in need of interventions aimed at increasing RTW self-efficacy, such as implementation of a graduated RTW plan, or communication of praise/highlighting of worker’s successes. As such, self-efficacy can be part of a screening intervention aimed at identifying early on workers at risk of prolonged work absence. Change scores can be very useful for those who are actively working with workers towards a RTW, later on in a claim trajectory, such as vocational rehabilitation consultants, clinicians, case managers, to identify those workers who are struggling with increasing their readiness for RTW or with remaining at work.

To measure self-efficacy regarding RTW, we developed a 10-item scale to assess self-efficacy of workers to return to work—the Return-to-Work Self-Efficacy (RTWSE) scale. This scale has three factors—ability to cope with pain, obtaining help from supervisor, obtaining help from co-workers. The structural and construct validity of the scale has been supported in a sample of injured workers with back and upper extremity MSK disorder, and published in an earlier paper [23].

The current study aims to examine the predictive validity of the RTWSE scale using the same cohort used for initial validation [23]. The current study examines the predictive validity of both the baseline absolute value of the RTWSE scores and the changes in RTWSE scores from 1-month to 6-month time points, as they relate to the RTW status at 6- and 12-month follow-up, in a sample of injured workers with MSK pain.

## Materials and Methods

### Study Design

This study was conducted within the sampling frame of the Readiness-for-RTW cohort [24, 25], a prospective cohort study of lost-time claimants with work-related low back or upper extremity MSK pain, who were recruited in cooperation with the Workplace Safety and Insurance Board (WSIB) of Ontario, Canada. A detailed description of the participant recruitment procedure has been published elsewhere [25]. Participants were interviewed by phone at 1-, 6-, 12- and 24 month post-injury. In this study, we used the 1-, 6- and 12-month follow-up data. The study was approved by the University of Toronto Ethics Review Board.

### Measurements

#### Return-to-Work Self-Efficacy Scale

Self-efficacy for RTW was assessed with the 10-item RTWSE scale [23] and measured at baseline and 6-month follow-up. This scale assesses self-efficacy for RTW within three subdomains: (1) the RTWSE Pain subscale, i.e. the ability to cope with pain (pain-tolerate, pain-prevent, pain-manage), (2) the RTWSE Supervisor subscale, i.e. the ability to obtain help from supervisor and (3) the RTWSE Co-workers subscale, i.e. the ability to obtain help from co-workers. Respondents rated their confidence for each item on a 5-point scale (1 = not at all certain, 5 = completely certain). Summative scores were calculated for the ‘Overall RTWSE’ scores and subscale scores, with linear transformation to give the same potential range for all scores from 2 to 10 with a higher score indicating better self-efficacy. The internal consistency was satisfactory for the overall self-efficacy scores (0.76) and for the subscales (ranging from 0.66 to 0.88) as previously reported [23].

RTWSE improvement was defined as an increase between baseline and 6-month follow-up scores. Two groups were created: Improved group—any increase (>0) on the RTWSE subscales at the 6-month follow-up compared to baseline, and a Not-improved group—the same or a lower score on the RTWSE subscales at 6-month follow-up compared to baseline.

### Confounders

Age and gender were based on self-report. Participants reported their marital status using five categories: married; single; widowed; living with a partner or common-law partner; separated or divorced. The variable was dichotomized (living with partner; living without partner) for the purpose of the analyses. Level of education and annual personal income were assessed through self-report as categorical variables.

Functional status associated with back pain was measured using the Roland–Morris Disability Questionnaire [26], a 24-item questionnaire assessing the presence of activity limitations. The Roland–Morris has been shown to have good psychometric properties [27, 28]. In the baseline sample, the internal consistency (Cronbachs α) was 0.92. The 11-item QuickDASH was used to assess functional status in participants with MSK disorders of the upper limb [29]. The QuickDASH is a shortened version of the DASH Outcome Measure [30]. Initial testing has shown that the QuickDASH has good psychometric properties [29]. The internal consistency in the present study was 0.90.

When participants reported pain in both the back and upper extremity, they completed both the Roland–Morris and the QuickDASH. For these participants, scores from each instrument were converted into a z-score and the highest z-score was used as the index of functional status. For participants completing only one measure of functional status, the z-score of that measure was used as the index of functional status. In addition, for those completing both measures, determination of the main pain site, was based on the highest z-score on the Roland–Morris or the QuickDASH.

Pain severity was assessed by taking the average of two items from the intensity subscale of the Von Korff Pain Scale—pain “right now” and average pain over the last month [31]. Both items are rated on a scale from 0 (no pain) to 10 (pain as bad as could be).

Depressive symptoms were measured with the 20-item Center for Epidemiologic Studies Depression (CES-D) scale [32]. The CES-D internal consistency was 0.92, measured in the baseline sample.

### RTW Status

Two RTW status groups were constructed (RTW, Not-RTW), based on the workers’ responses to the following yes/no question: “Are you currently working at any job right now?”. RTW status was assessed at baseline (1 month after the onset of sickness absence), 6- and 12-month follow-up. Workers were considered as having returned to work if they returned in any capacity—with or without limitations or restrictions.

### Statistical Analyses

Logistic regression analyses were performed to study the predictive validity of the RTWSE scale with RTW status as the dependent variable. Logistic regression was used since the outcome is binary, and logistic regression is a commonly used modelling choice for binary outcomes that allows to understand the relationship between RTWSE and the RTW outcome, while controlling for other variables.

The analyses were adjusted for age, gender, educational level, personal income, pain site, pain severity, functional status, and depressive symptoms, and for baseline RTWSE scores in the improvement score analyses. The purpose of the analyses was to examine self-efficacy and changes in self-efficacy as markers of future working status outcome, but not to generate an explanatory model. Self-efficacy is influenced by a number of factors—work climate, relationship with supervisor, physical and mental demands of job, as well as pain intensity and functional status. The purpose of the analyses was not to consider the relative contribution of these factors to the outcome, but focus on the role of self-efficacy.

The first step in the analysis was to examine unadjusted associations between RTWSE and RTW status. In the second step, we added the confounding variables. Different time windows were applied, investigating the predictive validity of RTWSE at baseline for RTW status at 6- and 12-month follow-up, and the predictive validity of improvement scores between baseline and 6-month follow-up for RTW status at 12-month follow-up. All statistical models were based on the (varying) number of people available at the three different waves. The analyses were carried out with the statistical package SPSS 18 (SPSS Inc. Released 2009, PASW Statistics for Windows, Version 18.0. Chicago, SPSS Inc).

## Results

### Participation Rates, Timing of Interviews, and Description of the Sample

A total of 632 claimants completed the baseline telephone interview 1 month post-injury with a participation rate of 61 %, consistent with participation rates of other cohort studies of adults with MSK conditions, which range between 55 % [33] and 63 % [34]. A detailed description of the flow of participants is found elsewhere [24, 25]. A total of N = 446 participants completed the 6-month interview with a retention rate of 71 %; N = 383 participants completed the 12-month interview, which resulted in a retention rate of 61 % of the baseline sample. At baseline, the sample consisted of 55.4 % male, 63.5 % aged ≥40 years and 66 % having back pain. Forty-four percent of the workers worked at the time of injury 37.5–40 h/week, 27 % worked >40 h/week. As well, a previous publication using the same baseline sample showed that at baseline 55.5 % of participants had received a work accommodation offer, with 73.2 % accepting the offer [25]. The sample socioeconomic characteristics are presented in Table 1. Means and standard deviations for baseline and 6-month RTWSE scores are found in Table 2, as well as means and standard deviations for baseline pain severity, CES-D scores, and functional status.

**Table 1:** Socio-demographic characteristics

Interview data	Follow-up					
	One month N = 632		Six months N = 446		Twelve months N = 383	
	*N*	(%)	*N*	(%)	*N*	(%)
Gender (male)	350	55.4	238	53.4	213	55.6
Age categories (years)			(N = 445)^a^		(N = 382)^a^	
15–29	93	14.7	56	12.6	44	11.5
30–39	137	21.7	78	17.6	67	17.5
40–49	228	36.1	167	37.8	146	38.2
≥50	173	27.4	144	32.0	125	32.7
Education						
Some high school	112	17.7	71	15.9	59	15.4
High school completed	177	28.0	122	27.4	98	25.6
Some post-secondary	130	20.6	92	20.6	89	23.2
Post-secondary/some graduate education	213	33.7	161	36.1	137	35.8
Personal income	(N = 596)^a^		(N = 423)^a^		(N = 367)^a^	
<$20,000	95	15.9	69	16.3	55	15.0
$20,000–39,999	240	40.3	170	40.2	142	38.7
$40,000–59,999	180	30.2	121	28.6	112	30.5
>$60,000	81	13.6	63	14.9	58	15.8
Pain site						
Back	418	66.1	283	63.5	263	68.7
Upper extremities	214	33.9	163	36.5	120	31.3
Number of hours worked at time of injury (h/week)						
≤37.5	179	28.3	142	31.8	120	31.3
37.5–40	281	44.5	192	43.1	160	41.8
≥40	172	27.2	112	25.1	103	26.9
Number of workers at worksite at time of injury (workers)	(N = 630)^a^		(N = 445)^a^		(N = 382)^a^	
<20	180	28.6	118	26.5	98	25.7
20–99	199	31.6	143	32.1	128	33.5
100–299	133	21.1	102	22.9	81	21.2
≥300	118	18.7	82	18.4	75	19.6
Self-reported work absence: number of work days missed at 1-month interview (SD) (median)	14.5 (7.1) (14.0)		14.3 (6.7) (14.0)		14.1 (6.8) (14.0)	
Self-reported work absence: number of work days missed at 6-month interview (SD) (median)			46.5 (53.4) (20.0)		46.1 (53.9) (20.0)	

^a^Some *n*’s are reduced due to missing data

**Table 2:** Means and SDs of RTWSE scores (baseline, 6-month and change) and of pain, functional status, and depressive symptoms (baseline)

	Baseline Mean (SD)	Six months Mean (SD)	Change Mean (SD)
Self-efficacy for RTW			
Overall (range 2–10)	7.5 (1.6)	7.3 (1.8)	−0.1 (1.6)
Pain (range 2–10)	6.5 (2.0)	6.7 (2.1)	0.2 (2.1)
Supervisor (range 2–10)	8.3 (2.1)	7.8 (2.5)	−0.5 (2.4)
Co-worker (range 2–10)	7.7 (2.3)	7.6 (2.4)	−0.1 (2.3)
Pain (range 0–10)	6.7 (1.9)		
Functional status (z-score)	0.1 (1.0)		
Depressive symptoms (range 0–60)	16.1 (1.8)		

An attrition bias analysis, comparing 6-month interview respondents (n = 446) to non-respondents (i.e. lost to follow-up) (n = 186), revealed non-respondents were more likely to have worked longer hours at time of injury, and to have specified “back” as their primary pain site [24]. Moreover, male non-respondents tended to be younger than male respondents, whereas in women, differences in age were not as apparent. Comparing 12-month interview respondents (n = 383) to non-respondents (i.e. lost to follow-up) (n = 249) revealed that non-respondents reported better mental health (SF12) and fewer depressive symptoms (CES-D) compared to respondents. Six months after injury, participants were more likely to be receiving wage replacement benefits for a longer duration and to have a higher rate of re-instatement of wage replacement benefits than non-participants.

### Predictive Validity of RTWSE Scale on RTW Status

The RTW rate was 74 % (n = 329/446) 6 months post-injury, and increased to 77 % (n = 294/383) 12 months post-injury. Table 3 presents logistic regression analysis results. The crude analyses show that 1-month Overall RTWSE and 1-month Pain RTWSE scores predict RTW status at 6- and 12-month follow-up. After confounding variable adjustment, Pain RTWSE only remains significantly related to the RTW status at 6-month follow-up, with a trend for Overall RTWSE to be associated with 6-month RTW status.

**Table 3:** Logistic regression analyses with the RTW Self-efficacy scale as predictor, and 6- and 12-month RTW status as outcomes

	Crude			Adjusted^a^		
	Beta	OR (95 % CI)	*P* value	Beta	OR (95 % CI)	*P* value
*Baseline RTW SE scores compared to 6-month RTW status (N* *=* *419)*						
Overall score RTWSE	0.194	1.21 (1.06–1.38)	0.004*	0.115	1.12 (0.97–1.30)	0.13
Pain RTWSE	0.230	1.26 (1.23–1.41)	<0.001*	0.184	1.20 (1.06–1.37)	0.005*
Supervisor RTWSE	0.088	1.09 (0.99–1.21)	0.081	0.047	1.05 (0.94–1.17)	0.41
Co-worker RTWSE	0.047	1.05 (0.96–1.15)	0.317	0.003	1.00 (0.91–1.11)	0.96
*Baseline RTW SE scores compared to 12-month RTW status (N* *=* *366)*						
Overall score RTWSE	1.181	1.20 (1.04–1.39)	0.015*	0.073	1.08 (0.91–1.27)	0.396
Pain RTWSE	0.132	1.14 (1.01–1.28)	0.029*	0.012	1.01 (0.88–1.16)	0.869
Supervisor RTWSE	0.100	1.10 (0.99–1.24)	0.079	0.065	1.07 (0.94–1.21)	0.322
Co-worker RTWSE	0.092	1.10 (0.99–1.22)	0.083	0.042	1.04 (0.93–1.17)	0.471

*Improved RTW SE scores compared to 12-month RTW status (N* *=* *366)*						
Reference = not-improved	Crude			Adjusted^b^		
	Beta	OR (95 % CI)	*P* value	Beta	OR (95 % CI)	*P* value
Overall RTWSE	0.349	1.42 (0.84–2.40)	0.193	0.655	1.92 (1.04–3.57)	0.038*
Pain RTWSE	0.175	1.19 (0.72–1.99)	0.502	0.494	1.64 (0.87–3.08)	0.124
Supervisor RTWSE	0.255	1.29 (0.72–2.31)	0.391	0.697	2.01 (0.97–4.14)	0.060
Co-worker RTWSE	0.454	1.56 (0.89–2.79)	0.119	0.887	2.43 (1.18–5.00)	0.016*

* Significant at *p* < 0.05

^a^Adjusted for age, gender, education level, marital status, personal income, pain site, pain severity, functional status and depressive symptoms

^b^Adjusted for age, gender, education level, marital status, personal income, pain site, pain severity, functional status and depressive symptoms and baseline RTW-SE scores

In unadjusted analyses, no significant relationship is found between improvements on RTWSE subscales and the RTW outcome at 12-month follow-up. However, after adjusting for baseline RTWSE scores and for confounding variables, improvements in Overall RTWSE and Co-worker RTWSE predict 12-month RTW status, with trends for improvements in Supervisor RTWSE and Pain RTWSE.

## Discussion

Our findings support the predictive validity of the RTWSE scale in claimants with MSK. The Pain RTWSE scores measured 1 month post-injury were useful to predict RTW status 6 months post-injury. There was a trend for baseline Overall RTWSE to be predictive of RTW status 6 months post-injury. To predict 12-month RTW status, improvements in Overall RTWSE and in Co-worker RTWSE were useful, with trends for improvements in Supervisor RTWSE and Pain RTWSE. In accordance with previous studies [16, 18, 19, 21], we found that SE demonstrated predictive validity in RTW outcome in work disabled workers; lower levels of baseline self-efficacy were associated with a lower likelihood of being back at work 6 months post-injury. In addition, self-efficacy regarding ability to manage pain at work was important in the early phases of workers’ RTW trajectory.

We found differences in predictive validity of dimensions of RTWSE over time. Baseline Pain RTWSE predicted (after adjustment) 6-month RTW status, while improvements in Overall and Co-worker RTWSE best predicted 12-month RTW status. Baseline self-efficacy did not predict 12-month RTW status. These findings point to the importance of incorporating strategies to enhance self-efficacy in RTW interventions, such as strategies to prevent re-injury and pain exacerbation, pain management, work accommodations, and strategies to address impact of co-workers. Differences in when the effect of predictive factors can be observed over the course of RTW trajectories have been reported before; phase-specific predictors have been considered by other researchers including Krause et al. [35], Dasinger [36] and Frank et al. [37] for low back pain and RTW outcomes.

Baseline Pain RTWSE subscale predicted 6-month RTW status. The importance of baseline Pain RTWSE is corroborated by previous research, which stresses the importance of positive Pain RTWSE for successful adaptation to chronic pain [38–40]. Lower self-efficacy, or the lack of belief in one’s own ability to manage pain, to cope and function despite persistent pain, has been found to be a significant predictor of the extent to which individuals with chronic pain become disabled and/or depressed [38, 41].

Twelve-month RTW status was predicted by improvements in self-efficacy instead of baseline RTWSE scores. These results might be explained by the idea that the baseline assessment was too distal in time to maintain its predictive ability to 12-month work status. We found improvement on the Co-worker RTWSE scores to be the strongest predictor of RTW status at 12-month follow-up. The ORs of the improved Pain RTWSE and Supervisor RTWSE were also in the expected directions, although not significant.

The greater relevance of Pain RTWSE for earlier RTW status, and the greater relevance of Co-worker RTWSE for the 12-month RTW status suggest that in the earlier phases of the RTW process for a MSK injury, physical recovery and associated pain may be most prevalent in injured workers’ experience, as they adjust to the experience of pain, which tends to be more intense shortly after the injury. As time goes by and as the focus starts to shift to interacting with the workplace, initiated by worker or employer, the quality of the interactions with co-workers may be more important in the injured workers’ experience. These phase-specific effects [42] are consistent with the emerging evidence that workplace culture is strongly associated with RTW outcomes [43–45].

Our findings suggest that readiness to return to work 12 months post-injury may depend more on the changes in self-efficacy regarding ability to access help from co-workers, than on the baseline RTWSE. Perhaps it is difficult for workers to accurately judge Co-worker RTWSE at baseline, and only through attempts to return to work or contemplating RTW are they able to reflect on that matter. Moreover, RTW at 12-month follow-up may depend more on the ability to access help at work than to manage pain symptoms.

This is the first study to evaluate the predictive validity of the 10-item RTWSE scale. In addition, it is one of the first studies to consider the predictive validity of improvements in self-efficacy over time with the RTW outcome. When interpreting the results, the following methodological issues must be considered. Though reasonable for a study among claimants, the overall participation rate of 61 % raises the question of selective participation, which may have biased the results. However, the cohort was shown to be representative of the most comparable claimant group with regards to basic demographic and workplace variables, but not with regards to duration of time receiving wage replacement benefits and rates of wage replacement re-instatement, suggesting the presence of more severe disability in the cohort [24].

A related issue concerns the loss to follow-up of 29 % (at 6-month follow-up) and 39 % (at 12-month follow-up). The attrition analysis demonstrated that at 12-month follow-up, non-respondents were younger males, and reported a better mental health condition and fewer depressive symptoms compared to respondents. As poor mental health and self-reported depressive symptoms have been known to be negatively associated with RTW status, our results at 12-month may differ with 6-month results due to attrition bias. However, when we re-analyzed the predictive validity of the baseline RTWSE (sub)scales for RTW status at 6-month follow-up, and we excluded those who became drop-outs at 12-month follow-up, we did not find any differences in our findings. Therefore, we expect that the drop-outs did not influence the 12-month results.

An improvement on the RTWSE scale was defined as any higher score on a RTWSE subscale at 6 months follow-up compared to baseline, no difference or a lower score on the RTWSE subscale was defined as ‘not-improved’. We recognize the use of the word ‘improvement’ implies a meaningful change. The purpose of this paper was to examine predictive validity, not minimally important differences. An examination of minimally important differences would require additional self-reported information or clinical information period. Unfortunately, this information is not available.

In conclusion, the study found evidence supporting the predictive validity of the RTWSE scale within 12 months post-injury, when controlling for important confounding socioeconomic, pain, functional status, and depression variables. The RTWSE scale may be a potentially valuable scale in research and in managing work disabled claimants with musculoskeletal disorders. Further research is needed to confirm the construct validity and predictive validity of the RTWSE scale in other populations, and to evaluate test–retest reliability and responsiveness. In addition, the role of changes in self-efficacy on development and duration of work disability should be further investigated.
